# Lemierre Syndrome: A Rare Complication of Infectious Mononucleosis

**DOI:** 10.7759/cureus.77443

**Published:** 2025-01-14

**Authors:** Sara D Tipmongkol, Colton M Hurley, James Eppelbaum, Edward C Owens

**Affiliations:** 1 Internal Medicine, Lake Erie College of Osteopathic Medicine, Erie, USA; 2 Internal Medicine, Meadville Medical Center, Meadville, USA; 3 Family Medicine, Meadville Medical Center, Meadville, USA

**Keywords:** anticoagulation, infectious mononucleosis, internal jugular vein abnormality, internal jugular vein thrombophlebitis, lemierre's, lemierre's-like syndrome, oropharyngeal infection, septic thrombophlebitis, streptococcus intermedius bacteremia, thrombotic vasculopathy

## Abstract

Lemierre syndrome is a rare condition that arises from septic thrombophlebitis most commonly in the internal jugular vein. Common precipitating factors include recent oropharyngeal, tonsillar, or dental infection or chronic bacteremia. While modern antibiotic usage has decreased mortality rates, challenges persist in accurately diagnosing the condition at the time of patient presentation due to a lack of awareness of this rare disorder. Here, we present the case of a previously healthy 20-year-old female patient who was diagnosed with infectious mononucleosis approximately two weeks before her hospital admission. She presented with a five-day history of shortness of breath and cough. Diagnostic workup/evaluation revealed pneumonia with bilateral pleural effusions as well as the need for intravenous antibiotics and oxygen via nasal cannula, prompting her admission to the hospital. During her hospitalization, blood cultures showed growth in two out of four test tubes with *Streptococcus intermedius*, a bacterium commonly associated with abscess formation. This finding led her medical team to conduct a computed tomography scan of her cervical region, revealing significant septic thrombophlebitis within her internal jugular vein.

## Introduction

Lemierre syndrome, a rare disease triggered by recent oropharyngeal infections, results in septic thrombophlebitis of the internal jugular vein and metastatic foci [1]. The pathogenesis of Lemierre syndrome involves mucosal damage of the pharynx caused by bacterial infection, which then spreads to the laryngeal space. This infection leads to thrombus formation, which is facilitated by both septic thrombi induced by the bacteria and increased platelet aggregation due to inflammation from infection [2]. If left untreated, this syndrome can lead to septic thrombi traveling to various organ sites such as the lungs, brain, and liver [3]. The majority of cases are attributed to Fusobacterium, a gram-negative anaerobic bacterium, which constitutes normal human microflora in the oropharynx, genitourinary tract, and gastrointestinal tract [4]. Afflicting one in one million people, this syndrome, if misdiagnosed, can lead to devastating consequences with a 90% mortality rate without treatment and even a 5%-18% mortality rate with antibiotic treatment [2,3]. Diagnosis relies on clinical presentation, increased suspicion, positive microbial culture, and characteristic radiological findings [5]. A focused exam of the neck, involving the suprasternal and supraclavicular region for signs of cellulitis, should also be performed and can indicate the spread of the infection beyond the internal jugular vein [2]. Treatment primarily focuses on resolving septicemia with antibiotics, with the possible need for surgical drainage [6].

## Case presentation

**Figure 1:**
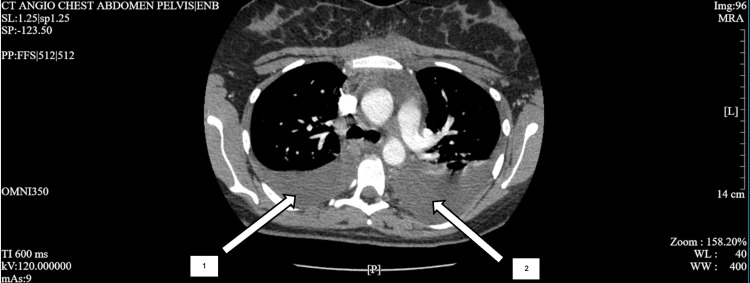
CT scan demonstrating bilateral pleural effusions taken upon admission Arrow 1 demonstrates a pleural effusion on the patient's right side and arrow 2 demonstrates a pleural effusion on the patient's left side CT: computed tomography

**Figure 2:**
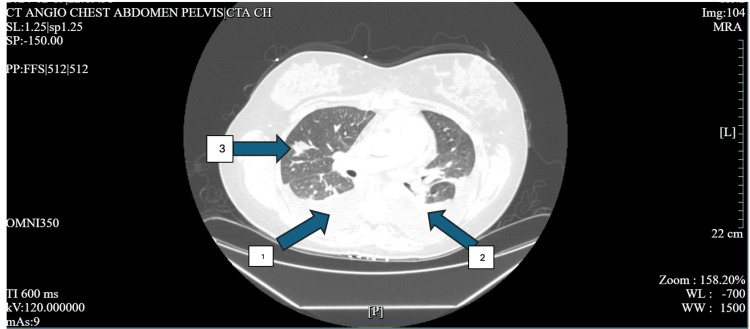
CT angiogram of chest with the right middle lobe infiltrate Arrow 1 demonstrates a right-sided pleural effusion, arrow 2 demonstrates a left-sided pleural effusion, and arrow 3 demonstrates a right middle lobe infiltrate CT: computed tomography

**Figure 3:**
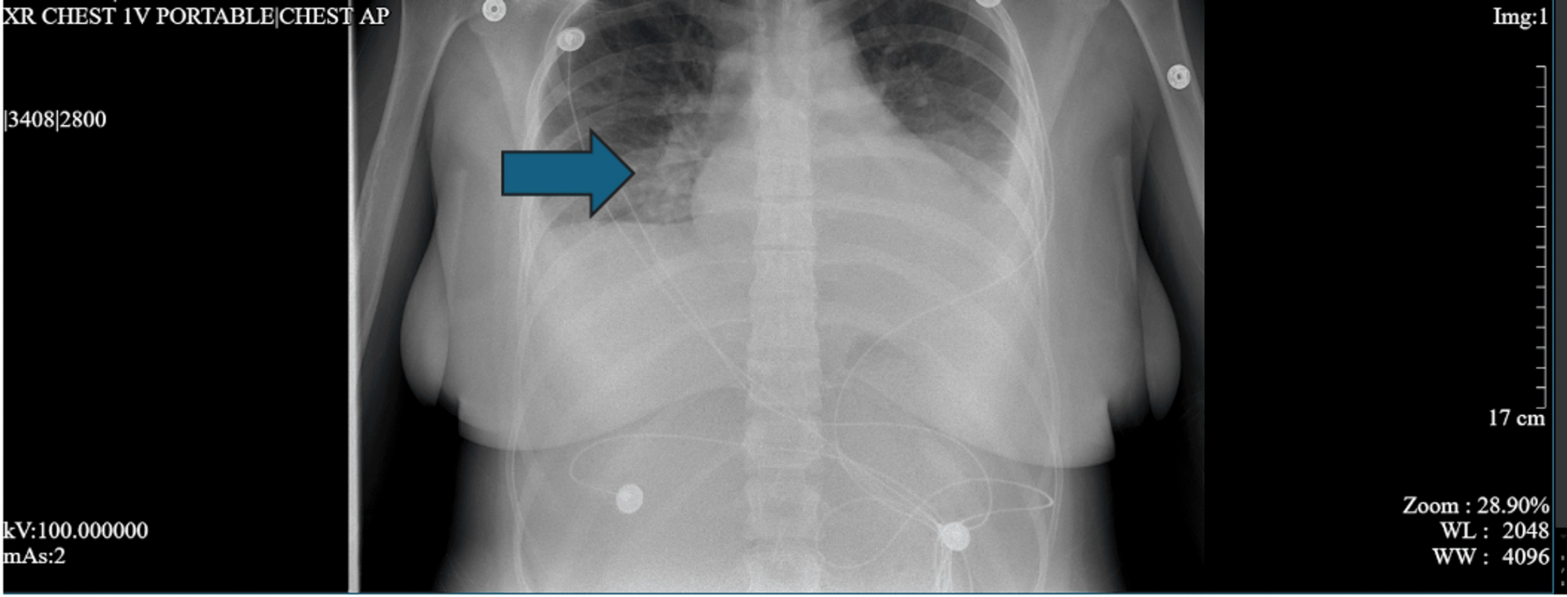
Chest X-ray taken on admission Arrow indicates the finding suggestive of right middle lobe consolidation

A previously healthy 20-year-old female patient presented to the emergency department with a five-day history of shortness of breath and cough. Additionally, she reported experiencing sharp pleuritic chest pain, nausea, vomiting, and recurrent fevers. Approximately 21 days before her emergency department visit, she had been diagnosed with infectious mononucleosis by the mono-spot test in UrgentCare. A physical examination revealed diminished lung sounds diffusely as well as tachycardia. The patient’s vitals upon arrival were as follows: heart rate: 147, blood pressure: 120/79, respiratory rate of 20, and oxygen saturation of 94%. The emergency department workup entailed ordering a chest X-ray, which revealed bibasilar infiltrates and bilateral pleural effusions. Additionally, a chest computed tomography (CT) angiogram revealed bilateral pleural effusions, pneumonia of the left lower lobe, as well as an infiltrate in the right middle lobe (see Figures 1-3). Her laboratory results demonstrated severely elevated inflammatory markers, with a procalcitonin of 75.24 ng/mL and an erythrocyte sedimentation rate of 55 mm/hour. Furthermore, her viral panel showed elevated levels of both anti-viral capsid antigen (VCA) immunoglobulin G and anti-VCA immunoglobulin M, which signified that she had a recent infection (see Table 1). This led us to the diagnosis of a coinfection of pneumonia and infectious mononucleosis. Blood cultures were drawn in two vials (aerobic and anaerobic) four minutes apart during her initial workup in the emergency department. In the emergency department, her oxygen saturation dropped to 88%, requiring 2 L of oxygen via nasal cannula. Due to her recurrent fevers, elevated white count, hypoxia requiring oxygen, and the need for intravenous (IV) antibiotics, she was admitted to the hospital for further investigation. Treatment was initiated with vancomycin and azithromycin, for suspected pneumonia while sputum cultures were taken to determine the pathogen responsible.

**Table 1:** Lab values upon admission through hospital stay WBC: white blood cell; ESR: erythrocyte sedimentation rate; PT: prothrombin time; INR: international normalized ratio; VCA: viral capsid antigen; IgM: immunoglobulin M; IgG: immunoglobulin G; CRP: C-reactive protein; pCO_2_: partial pressure of carbon dioxide

Labs with normal values	Admission	Day 2	Day 3	Day 4	Day 5
WBC (4.5-13.5 k/mm^3^)	13	12.6	12.6	14.1	7.3
ESR (1-19 mm/hour)	55	55	29	-	-
PT (9.6-13.9 seconds)	16.7	-	-	-	14.6
INR (0.9-1.2)	1.5	-	-	-	1.3
Anti-VCA IgM (<36 U/mL)	>160	-	-	-	-
Anti-VCA IgG (0-18 U/mL)	75	-	-	-	-
Fibrinogen (200-400 mg/dL)	-	-	-	-	656
D-dimer (150-230 ng/dL)	-	-	-	-	2,539
Lactic acid (0.5-2.0 mmol/L)	0.9	2.6	1.9	-	-
CRP (0.0-5.0 mg/L)	-	203.6	151.0	134.4	111.2
Procalcitonin (0.0-0.10 ng/mL)	75.24	47.14	58.91	32.48	20.71
Blood gas	-	-	-	-	-
pH (7.35-7.45)	-	7.56	7.58	-	-
pCO_2_ (35-45 mmHg)	-	26	24	-	-
Bicarbonate (22-26 mEq/L)	-	23.3	22.5	-	-

On day 2, she was increasingly hypoxic, and her blood gas results showed that she was in respiratory alkalosis, necessitating an increase in oxygen use from 2 to 5 L (Table 1). Cardiology was consulted on the same day, and a transesophageal echocardiogram was recommended for concern of endocarditis. The final results revealed no abnormalities in her myocardial structure or function except for a slight pericardial effusion in addition to her previously noted pleural effusions. Her oxygen requirement was successfully reduced back to 2 L as her oxygen demand declined. Pulmonology was also consulted due to the nature of her pleural effusions and oxygen dependency. They performed a thoracentesis to determine if there was an underlying etiology for the pleural effusions. Approximately 450 ccs of fluid were drained from the left hemithorax and sent for fluid analysis/cytology. The resulting thoracentesis showed no bacterial growth. The cell cytology revealed pleural lactate dehydrogenase of 105 international units/L, pleural total protein of 3.1 g/dL, and pleural albumin of 1.4 mg/dL (Table 2). These results confirmed an exudative effusion secondary to her pneumonia or prior infectious mononucleosis diagnosis. Toward the end of day 2, she continued to experience fevers but was able to return to room air.

**Table 2:** Flow cytology from thoracentesis LDH: lactate dehydrogenase; IU: international units

Flow cytology from thoracentesis	Values
Fluid neutrophils	60
Fluid lymphocytes	8.0
Pleural total protein (1-2 g/dL)	3.1
Total serum protein (6.4-8.3 gm/dL)	4.8
Pleural albumin (mg/dL)	1.4
Pleural LDH (IU/L)	105

**Figure 4:**
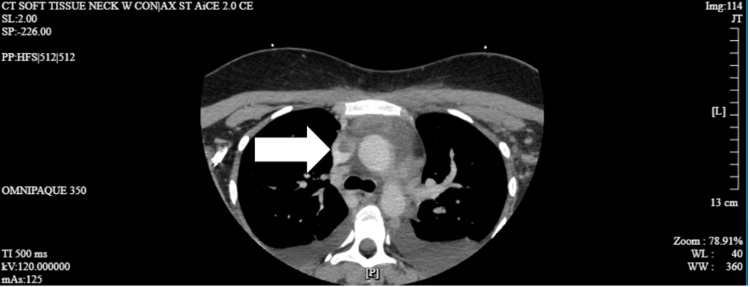
Septic thrombophlebitis in the internal jugular vein extending into the SVC (arrow) SVC: superior vena cava

On day 3 of her hospitalization, her inflammatory markers began to trend downward, but her white blood cell count remained elevated. At this point, one of the four blood culture bottles grew *Streptococcus intermedius* and *Micrococcus luteus*. She remained on her antibiotic regimen and exhibited signs of improvement. Consultation with infectious disease specialists confirmed the patient was on appropriate antibiotic therapy and required no additional agents. It was thought that the bacteria growing was a possible contaminant, and at this time, there was no concern or suspicion of Lemierre syndrome. By day 5, a second tube from her blood cultures taken upon admission also grew *S. intermedius*, raising suspicion of a previously unidentified underlying cause, particularly given this bacterium's association with abscess formation. Given that two out of four blood cultures were positive, along with the patient's lack of improvement, further investigations into other potential causes were conducted. A soft tissue CT was ordered to exclude a potential retropharyngeal/peritonsillar abscess. Additionally, a D-dimer and fibrinogen level assessment revealed significant elevations, indicating a substantial thrombus burden. After reviewing both the most recent soft tissue CT and the CT angiogram obtained upon her admission, a thrombus was found involving the left internal jugular vein, subclavian vein, and brachiocephalic vein, extending into the superior vena cava (Figure 4). Consequently, she was started on a heparin drip and her antibiotic regimen was broadened to include ertapenem per the recommendations of the infectious disease team. Pulmonology, infectious disease, and hematology specialists recommended her transfer to a tertiary care center to manage her significant thrombus burden, along with concerns for Lemierre syndrome. Upon discharge, her laboratory results showed significant improvement, with downtrending inflammatory markers and normalization of her white blood cell count.

Her heparin drip was continued during her transfer to a tertiary care center. During her inpatient stay, the patient's antibiotic regimen was changed due to recommendations from infectious disease from levofloxacin 750 mg to IV ampicillin-sulbactam to target more gram-positive bacteria, with plans to switch to linezolid 600 mg twice a day to complete the course at home. Upon transitioning home, the heparin drip was replaced with rivaroxaban. Vascular surgery was consulted and determined that direct intervention was unnecessary unless there were signs of acute decompensation in her clinical status. The patient was advised to take rivaroxaban for at least three months, with follow-up appointments scheduled with her primary care physician to determine the extent of treatment duration. Additionally, she was instructed to complete the course of linezolid for one month and discontinue oral contraceptives to decrease the risk of blood clots. About three months later, she had an intrauterine device placed and was advised to never go on estrogen-containing contraceptive therapy.

## Discussion

In most cases of Lemierre syndrome, the primary causative agents are *Fusobacterium necrophorum* and *Fusobacterium nucleatum*, both obligate anaerobic gram-negative bacilli. These bacteria are part of the normal oral flora and have gained attention for their potential role in other conditions, such as colon cancer and endometriosis [2,7,8]. Other pathogens, including Streptococcus spp., Bacteroides spp., *Staphylococcus aureus*, and *Klebsiella pneumoniae*, have also been identified as causative organisms [2,9,10]. In rare cases, *S. intermedius*, a normally benign oral bacterium, can be implicated. In our case, after a second blood culture grew *S. intermedius*, the infectious disease team ordered CT imaging to assess for abscess formation, as this organism is known to cause abscesses [11]. This led to the discovery of an internal jugular vein clot and the diagnosis of Lemierre syndrome.

Lemierre syndrome is characterized by septic thrombophlebitis, often associated with an inflammatory state. Initial imaging typically involves a chest X-ray to exclude other etiologies like peripheral infiltrates, nodules, or pleural effusions. Further imaging may include an ultrasound of the internal jugular vein to assess for thrombosis, followed by contrast-enhanced CT. Magnetic resonance imaging is considered the most sensitive modality for detecting septic thrombosis [2].

Although Lemierre syndrome caused by *S. intermedius* is rare, we present this case to highlight the importance of considering this organism in diagnosis. The incidence of Lemierre syndrome is reportedly increasing, though the reasons are unclear. Some factors that may contribute to this trend include increased awareness and antibiotic resistance, which limits treatment options [6]. Known risk factors for Lemierre syndrome include oropharyngeal trauma, Epstein-Barr virus infections, and hypercoagulable states [12]. As the condition predominantly affects younger individuals, it is crucial for healthcare providers to keep it in their differential diagnosis.

Treatment of Lemierre syndrome remains an area requiring further research, as there are no controlled trials to establish definitive treatment guidelines. Current therapy generally involves culture-sensitive antibiotics for six weeks to ensure adequate clot penetration [2]. Without treatment, mortality can exceed 90% [13]. Even with antibiotics, mortality rates range from 5% to 18%, with many patients requiring ICU admission and an average hospital stay of three weeks [2]. Complications can include pleural effusions, empyemas, pneumothoraces, pulmonary infiltrates, and septic thrombi, which can spread to the liver, kidneys, and brain [14,15].

The use of anticoagulation therapy in Lemierre syndrome remains controversial, with limited evidence supporting its benefit [16,17]. Some studies suggest anticoagulation may lead to hematoma formation or worsening of thrombosis, underscoring the need for individualized assessment. In most cases, anticoagulation is relatively safe, particularly in healthy patients with minimal comorbidities [18]. However, treatment decisions should be based on a careful evaluation of the patient's risk factors. For example, our patient was young with few comorbidities and a significant thrombus, so anticoagulation was considered to prevent the risk of stroke, especially given her oral contraceptive use. According to a case report by Lu et al., anticoagulation may be indicated if there is no improvement within 48-72 hours of starting antibiotics [19]. Ultimately, the decision to use anticoagulation must balance the benefits and risks, and should be tailored to each patient's unique situation.

## Conclusions

The primary focus of this work is to increase awareness of Lemierre syndrome. Although rare, it merits consideration in the differential diagnosis, as demonstrated in this case report. When confronted with a complex patient presentation that deviates from common patterns, it is vital to keep rare pathologies in mind. Effective interdisciplinary collaboration is essential for early diagnosis and proper treatment. Timely identification and intervention are critical in managing Lemierre syndrome, with antibiotic therapy being paramount in patient care. The dilemma of whether or not to utilize anticoagulation remains. Further research will be crucial in determining a more evidence-based standard of care.
